# Supplementary material for: Entelon (vitis vinifera seed extract) reduces degenerative changes in bovine pericardium valve leaflet in a dog intravascular implant model
Source: PLoS One. 2021 Mar 4;16(3):e0235454. doi: 10.1371/journal.pone.0235454 (PMC7932063; doi:10.1371/journal.pone.0235454)
Supplement: S1 Raw images — (PDF) [file pone.0235454.s001.pdf]

Original underlying images for all blot or gel data

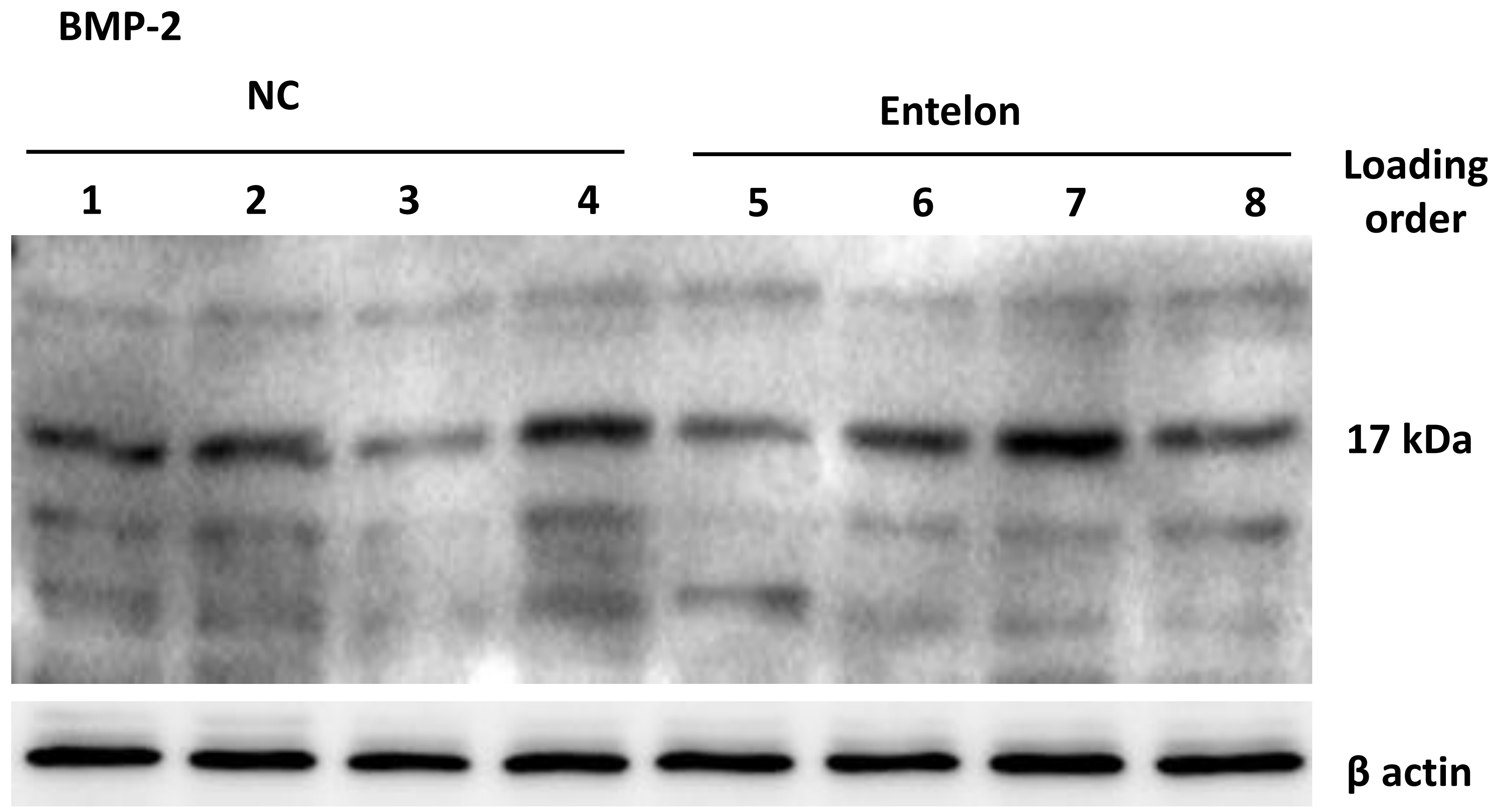
BMP-2
NC
Entelon
Loading order
1
2
3
4
5
6
7
8
17 kDa
β actin

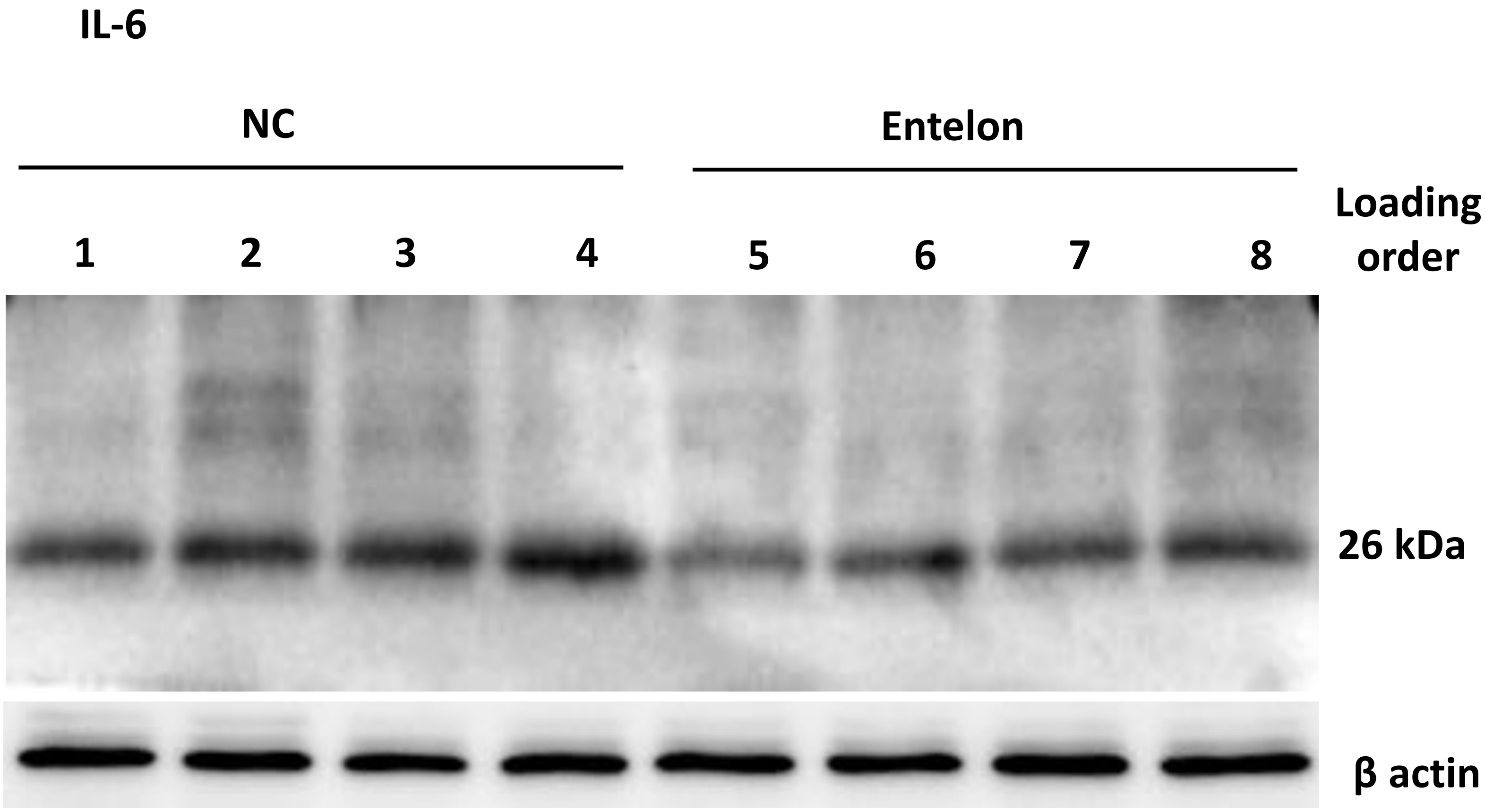
IL-6
NC
Entelon
1
2
3
4
5
6
7
8
Loading order
26 kDa
β actin

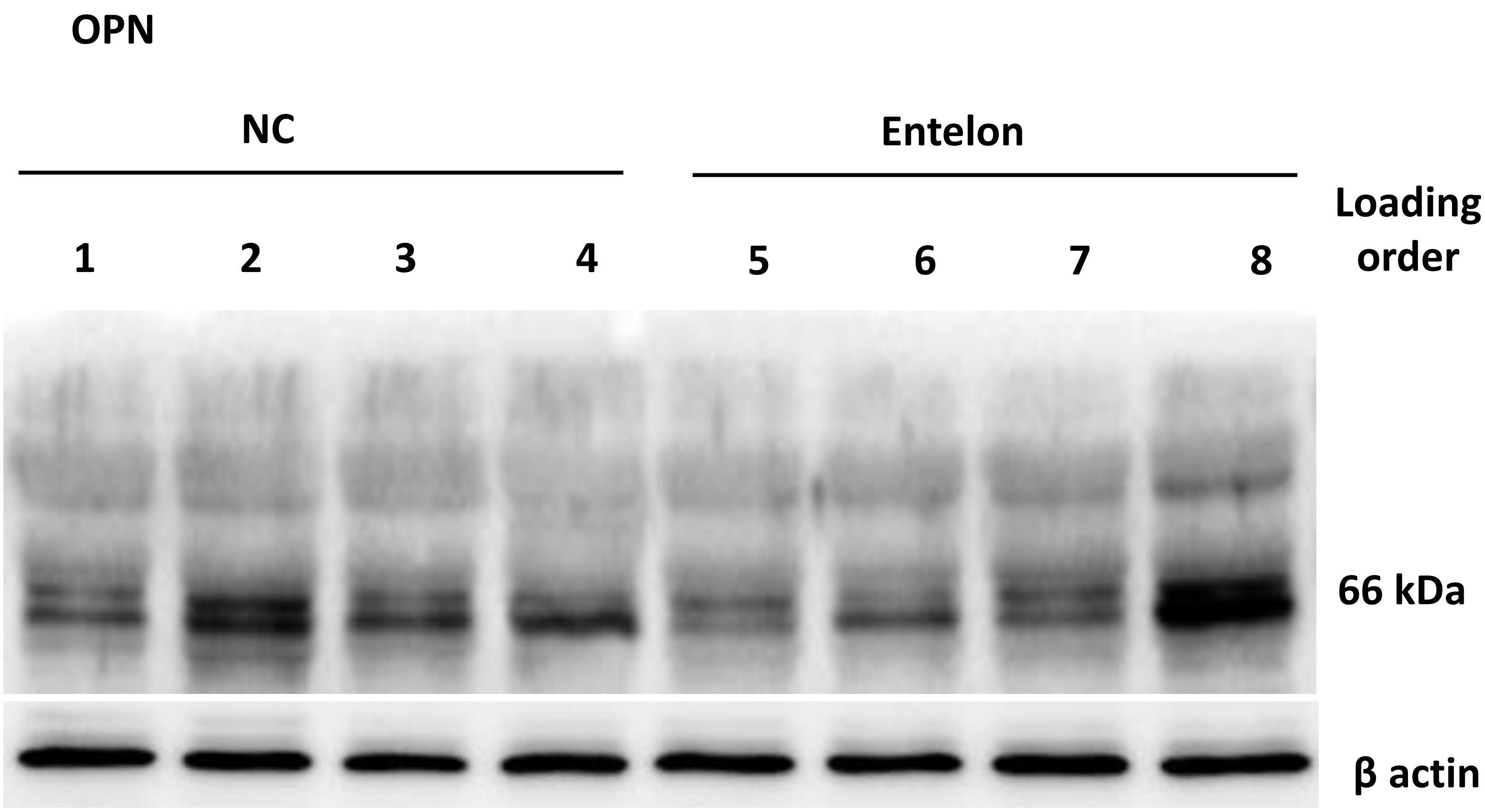
OPN
NC
Entelon
Loading order
1
2
3
4
5
6
7
8
66 kDa
β actin
